# Teaching breast cancer surgery in India: challenges and opportunities

**DOI:** 10.3332/ecancer.2022.1403

**Published:** 2022-05-26

**Authors:** Sanjit Kumar Agrawal, Piyush Ranjan, Noopur Priya, Shashank Nigam, Sumohan Chatterjee

**Affiliations:** 1 Department of Breast Oncosurgery, Tata Medical Center, Kolkata 700156, India; 2 Department of Surgical Disciplines, All India Institute of Medical Sciences, New Delhi 110029, India; 3 Department of Surgical Oncology, BALCO Hospital, Raipur 495684, India; 4 Private Oncology Clinic, Lucknow 226001, India; 5 Department of Breast Surgery, Manchester University Foundation NHS Trust, Manchester M239GP, UK

**Keywords:** breast cancer, training, India, surgery

## Abstract

Breast cancer (BC) incidence is increasing in India, and we need well-trained breast surgeons to deliver quality care to patients. However, BC surgery training in India is highly variable, evolving slowly and needs to be structured urgently. This article summarises the challenges and way forward for BC surgery training in India.

## Background

Breast cancer (BC) management has evolved significantly in the last 50 years, with better survival outcomes and improved survivors’ quality of life [1]. However, the estimated 10-year survival rate varies globally, with 83% in developed countries, 60% in middle-income countries and <40% in low-income countries [2]. The treatment opportunity and survival for BC patients depend on multiple factors, and BC surgery training is one of the essential factors. The 21st-century breast surgeon plays a central role in the multidisciplinary care team. The work responsibility has changed from mastectomy and axillary clearance to following initial diagnosis pathways, patient counselling, deciding the need for neoadjuvant therapy for downstaging or conservative surgeries [breast conservation surgery (BCS) and sentinel lymph node biopsy], oncoplastic breast surgery, therapeutic mammoplasty, localisation of the impalpable lesion, referral to genetic counselling and testing, prophylactic mastectomies, tumour board discussion and many more. Therefore, BC surgery training needs to be structured and multidisciplinary to enable clinicians to deliver the aforementioned complex treatment pathways to BC patients [3]. BC surgical training has been evolved and structured in developed countries with national university-recognised fellowships and degree courses [4].

BC is the most common cancer in India, with 1,78,361 estimated new cases in 2020 as per GLOBOCAN 2020 [1]. BC incidence has been increasing over the years in India, and we need qualified, trained BC experts to tackle the disease burden [5]. However, in India, the BC surgery discipline as a subspecialty training is slowly evolving and attracting young surgeons to choose this as a career in the last 10 years. Unfortunately, there are no published data for BC surgery training in India.

We aim to summarise the current training opportunity and future directions for BC surgery in India.

## Material and methodology

The summary is an opinion piece developed by meetings and discussions among authors. All but one author work in different parts of India with a high volume of BC surgical work and run training programmes for BC surgery. Additionally, a detailed web-based search was made to summarise the currently available resources for BC surgery in India. We have searched the National Medical Commission (NMC) curriculum for details of recognised degree courses available for surgical oncology and breast surgery training [6].

## Current educational resources

There are different types of BC surgery training opportunities available in India, like fellowships in oncoplastic breast surgery, as a part of surgical oncology degree courses and Master of Chirurgiae (MCH) in breast and endocrine surgery.

The breast surgery fellowship programmes are most widespread across the country and are provided by many prestigious institutions like Tata Memorial Hospital (Mumbai), Postgraduate Institute (Chandigarh), Tata Medical Centre (Kolkata), Max Super Speciality Hospital (New Delhi), Manipal Hospital (Bangalore) etc. [7, 8]. These programmes are mainly focused on the multimodality management of BC and are helpful for surgeons choosing BC surgery as a career path. In addition to BC management, the fellowship programmes encourage trainees for clinical audits and research.

Surgical oncology training is an NMC recognised programme available in Diplomate of National Board (DNB) and MCH degrees. Both programmes are for 3 years and include learning about surgical management of all cancers. BC surgery training is part of this programme, along with other organ cancers [9].

MCH in breast and endocrine surgery is a newly started super speciality branch. One can take admission in these super speciality courses by completing a Master’s in general surgery. Sanjay Gandhi Postgraduate Institute, Lucknow, and All India Institute of Medical Science, Delhi, are the presently designated institutes for admission for the course [10]. In addition to breast, these courses cover thyroid surgery, primary hyperparathyroidism surgery, pheochromocytoma, other adrenal tumours, multiple endocrine neoplasia syndromes, etc.

A Master of Surgery (MS) degree in oncoplastic breast surgery is an online course by the University of East Anglia. This course has a centre in India for face-to-face interactive discussions. The specialist team at Norwich medical school provides six modules and accreditation and can be used to build towards a master’s award in principles of breast oncoplasty. The course is designed to cover the clinical and surgical aspects. The learning is supported by formative assessments, critical thinking and decision-making. The application process can be directly made through the University of East Anglia [11].

The surgical oncology training is available across India. However, the specific BC surgical training opportunities (organ-specific fellowships) mentioned above are presently limited to tier-one metro cities of India.

## Challenges

The challenges faced in India for providing healthcare are daunting and they partly overlap with the challenges faced by surgeons providing breast care. The general surgeon manages a considerable burden of this ominous cancer along with specialised training. This trend has percolated down the generations and has, in a way, prevented breast surgery from becoming an exclusively organ-specific branch. That being said, the ever-growing magnanimous burden of BC demands an all-hands-on-deck approach. Therefore, the need of the hour is to train the ‘trained’ general surgeon to impart state-of-the-art breast care. This will ensure that all women receive individualised treatment at par with international standards and guidelines. It will also bring down the mastectomy rates in this country and promote BCS.

Another challenge rooted in this country is the lack of basic infrastructure and interdepartmental coordination. There are tertiary care setups without the facility of performing a sentinel lymph node biopsy and frozen section. These factors will haunt us, but they will also prevent us from providing world-class care for this disease. We have a short supply of doctors in the rural areas and lack a proper referral system. This has resulted in a false state of supply and demand mismatch. Certain government centres and big corporate setups are offering dedicated BC care. There is nothing in between to bridge these two ends depicting the wide cost gap. According to the studies, the current prevalence of BC in India stands at 25.8 per 100,000 population, against the general surgeon rate of 2.5 per 100,000 [12, 13]. The rate of general surgeons and surgical oncologists practising breast surgery is unknown, but surely not enough.

A significant part of breast surgery that our trained oncology colleagues often overlook is benign breast disease. It is not as ominous as BC but demands a specialised approach. The benefit of this will be two-pronged. Firstly, the clinical management of the condition and, more importantly, it will lead to more research work in the area, promoting novel and optimised therapeutic options rather than an symptomatic approach.

## Suggestions for the way forward

BC surgery training is highly variable and poorly regulated in India. The creation of breast surgery as a subspecialty is a long road and one with many obstacles, but a necessary one to take. There is a pressing need to assess the current human resources and the projected shortfall in the next 10 years in breast surgery. This is essential to plan for appropriate intervention, to deliver high-quality service providing workforce and to simultaneously manage increasing demand and patient expectations. A practical solution to improve the current situation and ensure a well-trained workforce is to introduce a national curriculum with objective endpoints and a short assessment to give a practising green light. It can be incorporated as an additional incentive and made mandatory in a phased manner.

The national curriculum should focus on the entire strata of the medical fraternity. Training basics should be laid in the undergraduate years and capitalised during the postgraduate years and beyond as a subspecialty. It should be a mixed model training with working in recognised units and centres of excellence as an apprenticeship, augmented with conferences and hands-on cadaveric and live workshops. The training should be structured, goal-oriented and documented with a logbook and work-based assessments during the postgraduate years and beyond. This will teach the practice of reflective and introspective learning among young surgeons. There should be regular appraisals for the practitioners to implement new operations and technology into their practice. The change we wish to see will take its due course with perseverance and, most importantly, inter-speciality coordination. The proposed framework is summarised in Table 1:

This study has some potential limitations. The study has pointed out the available training resources and gaps in breast surgery training in India. The proposed training module is the authors’ opinions and needs to be discussed with different stakeholders. The Association of Breast Surgeons of India, Asian Society of Mastology, Indian Society of Surgical Oncology and National Cancer Grid are a few of the eminently known organisations which are mainly focused on continuing medical education sessions and proposing guidelines for BC management as per the locally available resources [14, 15]. We need to devise a structured curriculum, and national BC societies should play a more prominent role to achieve this goal. European experts conducted a similar exercise recently to define the breast surgery training curriculum in European countries [4, 16]. The training should be equivalent and organ-specific to empower clinicians offering modern breast surgeries as per available resources.

## Conclusion

Breast surgery alone is not a recognised degree course in India and needs regulatory changes. Ongoing efforts by tertiary care cancer institutions with high-quality BC surgery fellowships are slowly pushing the breast surgery subspecialty formation likely soon. Urgent assessments of current human resources with the projected shortfall in the next 10 years are required to plan for an appropriate workforce to deliver high-quality service and to manage increasing demand and patient expectations.

## Conflicts of interest

The authors declare that they have no conflicts of interest.

## Funding

This study did not receive any specific grant from the public, commercial or not-for-profit funding agencies.

## Figures and Tables

**Table 1.** Proposed breast surgery training for career development.

Level	MBBS	Masters in general surgery	Super speciality training (Surgical oncology MCH/ DNB, fellowship programme)
Oncology basics	Basics breast anatomy, pathology, pharmacology	Management of benign and malignant BC Clinical audits	Comprehensive management of benign and malignant BC Multitumour board discussions Decision-making Clinical audits and research
Surgery component	Basic surgical skills	Level one breast surgery training (Lumpectomy, modified radical mastectomy)	Advanced breast surgery training (Axillary management, level one and two oncoplastic breast surgery and postmastectomy reconstruction)
Skills development	Clinical examination and diagnostic pathway of breast diseases Observation of breast surgical cases	Reinforcement of clinical examination and diagnostic pathway Independent and assisted breast surgery training	Reinforcement of clinical examination and diagnostic pathway Independent and assisted breast surgery training
